# Supplementary material for: A validity study to consult on a protocol of a home hazard management program for falls prevention among community dwelling stroke survivors
Source: PLoS One. 2023 Jan 11;18(1):e0279657. doi: 10.1371/journal.pone.0279657 (PMC9833545; doi:10.1371/journal.pone.0279657)
Supplement: S1 Table — (DOCX) [file pone.0279657.s002.docx]

**Table 1. Protocol of the quasi-experimental study**

| **Design** |  | Two arm, single-blind quasi experiment |
| --- | --- | --- |
| **Randomisation** |  | Not Applicable |
| **Blinding** |  | Single blinded- assessor blind |
| **Participants** | Inclusion | (i) 45 to 80 years old; (ii) diagnosed with first-time or recurrent stroke within 24 months; (iii) living in the community; (iv) undergoing outpatient rehabilitation; (v) slight disability to moderately severe disability according to the Modified Rankin Scale; (vi) able to walk for a minimum of 10 metres unsupported (with or without aid); (vii) has concerns about falling or had a post stroke fall; (viii) cognitively intact (score <8 on the 6-item Cognitive Impairment Test); (ix) able to speak and understand Malay or English. |
|  | Exclusion | (i) bed-bound; (ii) clinically diagnosed dementia according to ICD-11; (iii) diagnosed with psychiatric illnesses; (iv) diagnosed with aphasia; (v) medically unstable for example unstable angina or untreated fits; (vi) participants who had a prior home assessment and modification |
|  | Recruitment | Screening of potential participants will be conducted via the co-investigator at site. All interested participants will be contacted by the primary researcher and appointments were made. Study detail is explained in written and oral to the participants. Once consent was obtained, the participants were assigned to either the intervention or control group. |
| **Sample size** |  | A minimum of 12-18 participants per group. |
| **Location and setting** | Location for data collection | Three (3) hospitals: two for intervention group, and one for control group |
|  | Setting of intervention | Online and participant’s own home |
| **Data collection point** |  | Two (2) point: (i) pre intervention (baseline); (ii) post intervention. Conducted online via telehealth |
| **Outcome measures** | Screening | (i) Modified Rankin Scale; (ii) 6-Cognitive Impairment Test |
|  | Primary | (i) Home Falls and Accident Screening Test (HOME FAST); (ii) Falls Efficacy Scale- International (FES-I); (iii) Falls Calendar |
|  | Secondary | (i) Stroke Impact Scale 3.0; (ii) Canadian Occupational Performance Measure (COPM); (iii) Zarit’s Caregiver Interview; (iv) Short Form-12; (v) Feasibility Questionnaire (for intervention group only) |
| **Procedure** | Screening | At site (hospital) |
|  | Outcome Measures | Google or hardcopy forms |
| **Intervention procedure** | Experiment | (i) Results of the HOME FAST will be revealed to the intervention group with the suggested home modifications and assistive devices; (ii) education on falls prevention techniques, work simplification, energy conservation and home ergonomic will also be given.  Home modification / assistive devices provided: (i) grab bar for toilet and shower; (ii) double side tape to fix carpet or loose mats in place; (iii) storage box; (iv) torch light or one touch lights; (v) appropriate footwear; (vi) furniture riser; (vii) water scrapper; (viii) shower seat; (ix) toilet seat riser |
|  | Control | Continue usual care. Will receive home modification as in experiment after the study end. |
| **Duration of intervention** |  | 12 weeks |
| **Cost of intervention** |  | Approximately RM625 for equipment purchase and RM200 for installation per participant. |
| **Ethical approval** |  | MREC (NMRR-20-501-52933); JKEUPM (JKEUPM-2021-166) |
| **Trial registry** |  | ClinicalTrials.gov (NCT04618029) |
| **Funding** |  | Fundamental Research Grant Scheme (FRGS/1/2020/SS0/UPM/02/27), Ministry of Higher Education |
