# Supplementary material for: A validity study to consult on a protocol of a home hazard management program for falls prevention among community dwelling stroke survivors
Source: PLoS One. 2023 Jan 11;18(1):e0279657. doi: 10.1371/journal.pone.0279657 (PMC9833545; doi:10.1371/journal.pone.0279657)
Supplement: Attachment — Submitted filename: Response to Reviewers.docx [file pone.0279657.s003.docx]

Dear Reviewers,

Thank you for the comments and suggestions for our article. As below we have made amendments according to the suggestions.

Thank you.

| 5. Review Comments to the Author  The manuscript requires improvement based on the following comments.  Introduction  Line 172-195, the protocol section in the introduction is to be placed in the Materials and Methods section. | Protocol is placed in the Materials and Methods section (page 9, line 184) |
| --- | --- |
| Line 151, 207, 347, 361, there were numerous typographical errors e.g. applied, validate, attended, pilot. | Typographical errors were rectified |
| Line 179, the non-random method used to be stated e.g. how the subjects were assigned to the groups. | Clarification:  *Stroke survivors will be enrolled either in an experimental or control group according to the hospital of recruitment (page 9, line 191)* |
| Line 183, to state clearly the control group receives usual care only. | Rectified as suggested.  *Meanwhile, the control group will only receive standard care (page 9, line 196)* |
| Line 194, is this study, feasibility or pilot study? | The protocol is a feasibility study and pilot quasi-experimental trial.  *As this will be the first study to examine falls and home hazards among the stroke population, a feasibility study and pilot quasi-experimental trial would be the most appropriate study design as such practice has not been explored in Malaysia (page 8, line 175)* |
| Materials and methods  It is not clear whether the content of all the tools is in English or local version. Since Malaysia consist of various ethnicity, will this protocol be made available in the local language if the current protocol is in the English version? | *All of the outcome measures have been translated into the local languages namely Bahasa Melayu, Mandarin and Tamil, and all translated versions of the outcome measures have been validated* (page 9, line 201) |
| The actual protocol/content is to be included in the manuscript as appendices/supplementary. | Protocol is as supplementary file |
| The statistical software used in the analysis to be stated. | *Statistical Package for Social Sciences (SPSS) version 22 was used for data analysis (page 12, line 256)* |
| The rationale of having occupational therapists in the study evaluation and not involving other healthcare professionals such as occupational/rehabilitation/community/public health/geriatric doctors, hospital management staff etc or even caregivers is to be clearly stated. | *Only occupational therapists are selected to participate as previous studies indicated home assessments and modifications are typically conducted by occupational therapists and yield better outcomes [27] (page 10, line 222)* |
| Focus Group Discussion (FGD) could be employed in the study. | *For future studies, it is recommended that the protocol be reviewed qualitatively via focus group discussions of other healthcare professionals that are involved in falls preventions of stroke survivors as well as professional contractors or architects that have experience in providing home modifications for special populations (page 19, line 407)* |
| More samples could be recruited to strengthen the protocol especially with regards to validation aspect. | An extra 4 respondents were recruited to review the protocol.  *A total of 16 occupational therapists reviewed and rated the proposed intervention (page 12, line 261)* |
| Table 1, the sample size could be calculated (if it is a pilot study) and the study design to be stated. | *Hence, the sample size is considered optimal as suggested by Julious et al. [89] of between 12 and 18 participants per group for a pilot study (page 18, line 370)* |
| Results  Line 273, the Cronbach alpha values to be presented. | *The internal consistency for relevance of all the items had a Cronbach Alpha value of α=0.94 while for feasibility, the Cronbach Alpha value was α=0.97 (page 14, line 292)* |
