# Supplementary material for: A validity study to consult on a protocol of a home hazard management program for falls prevention among community dwelling stroke survivors
Source: PLoS One. 2023 Jan 11;18(1):e0279657. doi: 10.1371/journal.pone.0279657 (PMC9833545; doi:10.1371/journal.pone.0279657)
Supplement: Attachment — Submitted filename: Author_Response_to_reviewer_PONE-D-22-1767.docx [file pone.0279657.s004.docx]

Thank you for the opportunity for us to revise our manuscript. Here is our response on the comments.

| **Journal requirement** | |
| --- | --- |
| Please review your reference list to ensure that it is complete and correct. If you have cited papers that have been retracted, please include the rationale for doing so in the manuscript text, or remove these references and replace them with relevant current references. Any changes to the reference list should be mentioned in the rebuttal letter that accompanies your revised manuscript. If you need to cite a retracted article, indicate the article’s retracted status in the References list and also include a citation and full reference for the retraction notice. | We have re-checked the reference list and ensured there is no retracted paper cited in our manuscript. There is no new reference included in the current revision. |
| **Reviewer 2** | |
| Thanks for your efforts in editing the previous comments. Only the title of your manuscript should include the type of the study | We have modified the title by mentioning that this is a validity study of an intervention study protocol.  Full title from ‘Consultation on the relevance and feasibility of a home hazard management program for falls prevention among community dwelling stroke survivors’ was changed to ‘A validity study to consult on a protocol of a home hazard management program for falls prevention among community dwelling stroke survivors’  Short title from ‘Feasibility of a home hazard management program for falls prevention in stroke’ was changed to ‘Validity study of a home hazard management program for falls prevention in stroke’  In consequence, we have corrected the sub-title in our main manuscript to align with the corrected title:   - ‘Relevance and feasibility’ was changed to ‘Protocol Validity’ (p.12). |
